# Association of primary-site surgery with survival in patients with metastatic gastroenteropancreatic neuroendocrine carcinoma: a population-based SEER study (2004–2023)

**DOI:** 10.3389/fonc.2026.1910869

**Published:** 2026-07-22

**Authors:** Longlian Deng, Lemuge Che, Tao Liu, Zhaoliang Xie, Jing Yu, Yonglin Liu, Yu Liu, Qiang Xu

**Affiliations:** 1 Department of Abdominal Oncology, the Second People’s Hospital of Neijiang, Neijiang, China; 2 Department of General Internal Medicine, Hinggan League People’s Hospital, Ulanhot, China; 3 Department of Urology, West China Hospital, Sichuan University, Chengdu, Sichuan, China

**Keywords:** distant metastasis, neuroendocrine carcinomas, primary tumor surgery, prognosis, propensity score matching

## Abstract

**Background:**

Evidence regarding the association between primary tumor surgery and survival in metastatic gastroenteropancreatic neuroendocrine carcinoma (GEP-NEC) remains limited, and whether this association varies across patient subgroups is unclear. This study evaluated the associations between primary tumor surgery and overall survival (OS) and cancer-specific survival (CSS), and explored potential heterogeneity in effects.

**Methods:**

Patients diagnosed with distant metastatic GEP-NEC between 2004 and 2023 were identified from the SEER database and classified according to receipt of primary tumor surgery. One-to-one propensity score matching (PSM) was used to balance demographic, clinicopathological, and treatment-related variables. OS and CSS were compared using Kaplan-Meier analysis and Cox proportional hazards models. Sensitivity analyses, E-value analysis, and subgroup interaction analyses were performed to assess robustness and treatment-effect heterogeneity.

**Results:**

Among 4, 033 eligible patients, 786 underwent surgery, and 3, 247 did not. After PSM, 353 patients remained in each group. In the matched cohort, surgery was associated with longer median OS (26.0 vs. 8.0 months, P<0.001) and CSS (29.0 vs. 9.0 months, P<0.001). In the fully adjusted Cox model, surgery was associated with lower all-cause mortality (HR = 0.47, 95% CI: 0.40-0.56, P<0.001) and cancer-specific mortality (HR = 0.46, 95% CI: 0.39-0.54, P<0.001). Results were consistent across sensitivity analyses and remained robust in E-value analysis. Subgroup analyses showed stronger surgery–survival associations among patients who did not receive chemotherapy and, for CSS, among those with tumors <50 mm.

**Conclusion:**

Primary tumor surgery was associated with longer OS and CSS in selected patients with distant metastatic GEP-NEC, particularly those without chemotherapy or with smaller primary tumors.

## 1 Introduction

Neuroendocrine neoplasms (NENs) are a group of tumors originating from peptidergic neurons and neuroendocrine cells, characterized by high heterogeneity. Neuroendocrine carcinoma (NEC) is a poorly differentiated, highly malignant, and aggressive tumor type among NENs, with actively proliferating tumor cells and rapid disease progression (1, 2). Approximately 60-70% of NEC cases arise from the gastroenteropancreatic tract, and about 60% of patients have distant metastases at the time of initial diagnosis (3, 4). The prognosis of patients with NEC combined with distant metastasis is generally the worst among NENs, and their median survival time is often less than 1 year (3, 5). Because metastatic gastroenteropancreatic neuroendocrine carcinoma (GEP-NEC) was rare, high-quality evidence defining an optimal treatment model was lacking. Existing guidelines and expert consensus generally recommend platinum-based systemic therapy as the first-line treatment for advanced or unresectable NEC. They also emphasized that individual decisions should have incorporated tumor differentiation, the Ki-67 index, tumor burden, symptoms, performance status, and a multidisciplinary assessment (3, 6).

In recent years, numerous studies have shown that palliative surgeries significantly prolonged survival in patients with gastroenteropancreatic neuroendocrine tumors (GEP-NETs), including resection of the primary tumor and removal of metastatic lesions (7, 8). However, survival differed markedly between poorly differentiated NEC and well-differentiated neuroendocrine tumors (NETs). Guidelines generally did not recommend surgery for metastatic GEP-NEC, but those recommendations were based on limited studies, including a case report (9). Because GEP-NEC was rare, well-designed, large-sample studies were scarce to support the impact of primary tumor resection on survival in metastatic GEP-NEC. In recent years, however, an increasing number of real-world investigations have focused on the potential survival benefit of primary lesion surgery in NEC. A retrospective cohort study of United States stage IV GEP-NEC patients reported that primary or metastatic surgery lowered the risk of death by 40%, and combined primary plus metastatic surgery lowered it by 59%, compared with no surgery (10). Another retrospective study of patients with gastrointestinal neuroendocrine carcinoma (GI-NEC), accompanied by liver metastases, in whom hepatic metastatic lesions were not resected, showed that primary tumor resection significantly prolonged median OS (25.7 vs. 9.3 months) and was associated with a 57% reduction in the risk of death (11). These studies suggest that, even in the context of distant metastasis, selected patients with GEP-NEC may still derive benefit from primary tumor resection. However, the existing evidence has several limitations, including the inclusion of narrowly defined populations involving either primary or metastatic lesions alone, and study cohorts in which the years of diagnosis mostly ended in 2017 or 2018. These studies, therefore, may not fully reflect real-world outcomes after recent changes in diagnostic criteria, surgical techniques, and multimodal treatment strategies.

It is therefore necessary to further investigate the survival benefit of surgery in patients with GEP-NEC using more recent data. Based on recently updated SEER data, this study included patients diagnosed with distant metastatic GEP-NEC between 2004 and 2023 and systematically evaluated the association between primary tumor resection and OS and CSS. The aim was to provide updated population-level evidence regarding the potential survival benefit of primary tumor surgery in patients with metastatic GEP-NEC.

## 2 Materials and methods

### 2.1 Research design and study population

This population-based retrospective cohort study was a secondary analysis of SEER data from 2004 to 2023. Clinical, pathological, and treatment data for 18, 258 patients with GEP-NEC were extracted from the SEER Research Database of 17 Registries (2000-2023) using the National Cancer Institute (NCI) SEER*Stat software (version 9.0.43.0). Eligible GEP-NEC cases were identified from SEER using the variables “Site recode ICD-O-3/WHO 2008, ““Primary Site - labeled, “ and “ICD-O-3 Histology/Behavior.”Primary tumor sites were limited to the stomach, small intestine, colon and rectum, and pancreas. Specifically, the topographic codes included are C16.0–C16.9 (stomach), C17.0–C17.9 (small intestine), C18.0 and C18.2–C18.9 (colon), C19.9 (rectosigmoid junction), C20.9 (rectum), and C25.0–C25.9 (pancreas). Neuroendocrine carcinoma of the appendix has relatively unique biological behavior, so appendix site code C18.1 was not included in this study. NEC is defined using ICD-O-3 histology/behavioral code 8246/3: Neuroendocrine carcinoma, NOS. (**Supplementary Table 2**).

Patients were sequentially excluded according to prespecified Population, Intervention, Comparator, Outcome, and Study design (PICOS) criteria. The exclusion criteria were as follows: age younger than 18 years or 90 years or older; absence of microscopic confirmation or unknown diagnostic confirmation; multiple primary tumours during the patient’s lifetime; primary tumour located in the appendix; absence of distant metastatic disease; unknown or missing surgical status; surgery performed for non-primary lesions; missing survival or follow-up data; unknown race; and unknown residential environment.

**Figure 1:**
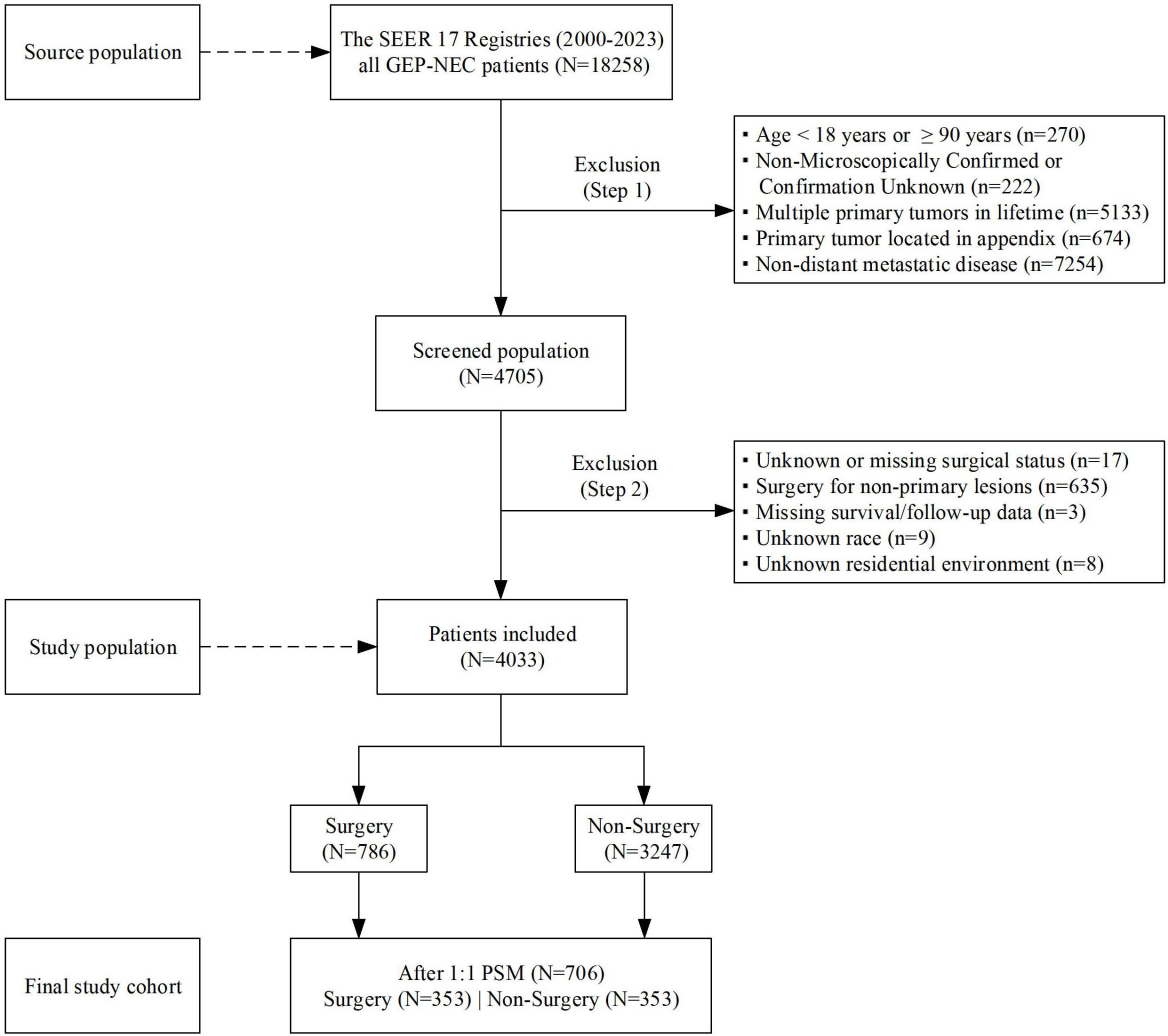
Flowchart of patient selection. GEP-NEC, gastroenteropancreatic neuroendocrine carcinoma; PSM, propensity score matching; SEER, Surveillance, Epidemiology, and End Results.

The patient selection process is shown in **Figure 1**. This study was reported in accordance with the Strengthening the Reporting of Observational Studies in Epidemiology (STROBE) guidelines (12). A complete list can be found in **Supplementary Table 1**. Because SEER is a publicly available, de-identified database, researchers who obtain official SEER access are generally exempt from additional institutional review board approval and informed consent requirements.

### 2.2 Exposure factors

Surgical intervention was the primary exposure in this study. Surgical status was defined as whether a patient underwent surgery at the primary tumor site. On this basis, patients were classified into the surgery and non-surgery groups. Surgical intervention included tumor ablation, tumor resection, and other procedures performed on the primary lesion for which the specific surgical type was unspecified. In brief, codes 00/A000 were classified as no surgery; codes 10–19/A100–A190 as tumor destruction or ablation; codes 20–80/A200–A800 as surgical resection; and codes 90/A900 as Surgery, NOS. Codes indicating unknown or missing surgery information were excluded from the analysis (**Supplementary Table 3**).

### 2.3 Primary and secondary endpoints

In this study, OS was the primary endpoint, and CSS was the secondary endpoint. OS was defined as the time from the date of diagnosis to death from any cause. Survival status was determined based on the “Vital Status recode (study cutoff used)” field in the SEER database. If a patient’s last follow-up date was later than the study cutoff date, the patient was considered alive. If the last follow-up date was on or before the study cutoff date and the patient was recorded as deceased, this was considered a death event. CSS was defined as the time from diagnosis to death from the cancer of interest. As only patients with a single primary tumor were included, the “SEER cause-specific death classification” variable was used for CSS analysis: deaths attributed to the cancer were treated as events, while deaths from other causes and alive patients were censored.

### 2.4 Covariates

The covariates included in this study were age (≤60 years/>60 years), sex (male/female), race (White/Black/other), type of residence (urban/rural), tumor site (stomach/small intestine/colorectal tract/pancreas), T stage, N stage, radiotherapy, and chemotherapy. In addition, this study ultimately included patients with GEP-NEC diagnosed from 2004 to 2023, a period during which the tumor-node-metastasis (TNM) staging system underwent substantial changes. From 2004 to 2015, the American Joint Committee on Cancer (AJCC) sixth-edition TNM staging criteria were used. The years 2016–2017 represented a transition period, during which the SEER Combined Stage was used. This system is based on the AJCC seventh-edition TNM staging criteria. This transition occurred because, for cancer cases diagnosed on or after January 1, 2016, United States cancer registries shifted from collecting staging information using the Collaborative Staging (CS) system to the TNM classification. The TNM classification, stage groups, and definitions used by SEER are based on the seventh edition of the TNM Classification of Malignant Tumors. From 2018 to 2023, the Extent of Disease (EOD) 2018 staging system was implemented, which is fully aligned with the AJCC eighth-edition staging criteria.

Because the rules defining TNM staging were not entirely consistent across these three periods, cross-version staging conversion may introduce subjective bias and misclassification. Therefore, this study directly used the T and N staging categories recorded in the original database as study variables. The year of diagnosis (2004-2015/2016-2017/2018-2023) was also incorporated into the regression model to adjust for potential confounding from changes in the staging system and temporal differences in diagnostic and therapeutic practices. This study included patients with primary malignant tumors of gastroenteropancreatic sites, including the stomach, small intestine, colorectal tract, and pancreas. Surgical information was derived from the North American Association of Central Cancer Registries (NAACCR) data item “RX Summ-Surg Prim Site (NAACCR #1291).” Notably, the coding system for this data item changed in 2023. Two- or three-digit numeric codes (for example, 00, 30, and 50) were used from 1998 to 2022, whereas four-digit alphanumeric codes (A000, A300, and A500) have been used since 2023. However, the surgical definitions corresponding to each code remained entirely consistent. To ensure continuity in the analysis of temporal trends, this study converted the 2023 alphanumeric codes back to their corresponding numeric codes for unified analysis.

### 2.5 Handling of missing values

T stage, N stage, primary tumor site, and tumor size are all clinically relevant prognostic factors in patients with GEP-NEC. Excluding patients with missing values for these key variables may introduce selection bias, reduce the available sample size, and decrease statistical power. Therefore, patients with missing values for these variables were not excluded. Cases with unavailable staging information were classified into the TX and NX subgroups, indicating unknown T and N stage, respectively. Tumor size was categorized as <50 mm or ≥50 mm based on the median of 50 mm, with an additional independent category for unknown tumor size. All unknown categories were included in subsequent regression models to adjust for potential confounding. Cases with missing values for other non-core study variables were excluded from the analysis. Sensitivity analyses were additionally performed using complete-case analysis, in which all cases with missing values were excluded, and multiple imputation based on random forests.

### 2.6 Sample size and power

Sample size and statistical power were estimated using Power Analysis and Sample Size (PASS) software (version 15.0). Because this was a retrospective cohort study, no prospective sample-size calculation was performed before data collection. Based on previous literature, with a statistical power of 0.90, a two-sided significance level of α = 0.05, an expected HR of 0.50 (13), and an assumed 20% loss-to-follow-up rate, the minimum required sample size was 87 patients per group. This requirement was substantially lower than the final matched sample size in the present study, which comprised 353 patients per group.

In addition, based on the adjusted HR from the multivariable Cox proportional hazards model in this study (HR = 0.46), the final sample size of 353 patients in the surgery group and 353 patients in the non-surgery group, and a two-sided significance level of α = 0.05, the estimated statistical power was 1.00 (100%). These results indicate that the sample size was sufficient for the planned survival analysis and that the risk of a false-negative result due to inadequate sample size was low.

### 2.7 Statistical analysis

All statistical analyses were performed using R software (version 4.4.2; https://www.r-project.org/). Normally distributed continuous variables are presented as the mean ± standard error of the mean (SEM), and between-group comparisons were performed using Student’s t-test. Non-normally distributed continuous variables are presented as medians with interquartile ranges (IQRs), and between-group comparisons were performed using the Mann-Whitney U rank-sum test. Categorical variables are expressed as frequencies and percentages, and between-group comparisons were performed using Pearson’s chi-square test. A 1:1 PSM method was used to match patients in the surgery group with those in the non-surgery group. This approach was used to reduce selection bias and minimize the influence of baseline differences in demographic and clinical characteristics on study outcomes. Matching factors included sex, age, race, tumor site, radiotherapy, chemotherapy, T stage, N stage, tumor size, and year of diagnosis. The matching ratio was 1:1, with a caliper value of 0.05. Between-group balance after matching was assessed using the standardized mean difference (SMD), with an SMD <0.1 indicating adequate balance between groups (**Supplementary Figure S1**, **S2** in the **Supplementary Material**). Because year of diagnosis remained slightly imbalanced after PSM, it was further included in the multivariable Cox regression models to reduce potential residual confounding. Multiple sensitivity analyses were also conducted to assess the robustness of the findings. The Kaplan-Meier method was used to plot OS and CSS curves, and the log-rank test was used to compare survival differences between the surgery and non-surgery groups. Multivariable Cox proportional hazards regression models were used to evaluate the association between surgery and OS or CSS in patients with GEP-NEC. Subgroup analyses were performed for all covariates, and interaction tests were used to assess whether the associations between surgery and survival varied across covariate subgroups. Several sensitivity analyses were performed to assess the robustness of the main findings: (1) analysis of the pre-PSM cohort; (2) analysis of the cohort excluded after PSM; (3) complete-case analysis, in which cases with missing or unknown T stage, N stage or tumor size were excluded; (4) analysis after multiple imputation of missing or unknown data; and (5) repeated PSM analyses using a relaxed caliper value of 0.2. In addition, the E-value was calculated to assess the potential impact of unmeasured confounding on the study outcomes. The E-value quantifies the minimum strength of association that an unmeasured confounder would need to have with both the exposure and the outcome to explain the observed exposure-outcome association fully. All tests were two-sided, with a significance level of α = 0.05. A P value <0.05 was considered statistically significant.

## 3 Results

### 3.1 Clinical characteristics of GEP-NEC patients before and after PSM

A total of 4, 033 patients with GEP-NEC and distant metastasis were included in this study, comprising 786 in the surgery group and 3, 247 in the non-surgery group. Before matching, baseline clinical characteristics differed significantly between the two groups. Compared with the non-surgery group, the surgery group had higher proportions of female patients, patients diagnosed between 2004 and 2015, primary tumors located in the small intestine or colorectal region, tumors <50 mm, T3/T4 disease, and lymph node-positive disease. In contrast, the median age, proportions of primary tumors located in the pancreas or stomach, and rates of radiotherapy and chemotherapy were lower in the surgery group than in the non-surgery group. These between-group differences were statistically significant (all P <0.05) (**Table 1**).

**Table 1:** Clinical characteristics of GEP-NEC patients in the surgery group and non-surgery group before and after PSM.

Characteristics	Before PSM			After PSM		
	Non-surgery (n=3247)	Surgery (n=786)	P value	Non-surgery (n=353)	Surgery (n=353)	P value
Gender (%)			0.002			0.651
Female	1367 (42.10)	378 (48.09)		162 (45.89)	168 (47.59)	
Male	1880 (57.90)	408 (51.91)		191 (54.11)	185 (52.41)	
Age, years (median [IQR])	63.00 [18.00]	62.00 [19.00]	0.003	60.00 [19.00]	61.00 [20.00]	0.126
Age (%)			0.027			0.821
≤60 years	1379 (42.47)	368 (46.82)		177 (50.14)	174 (49.29)	
>60 years	1868 (57.53)	418 (53.18)		176 (49.86)	179 (50.71)	
Race (%)			0.547			0.569
White	2589 (79.74)	640 (81.42)		284 (80.45)	273 (77.34)	
Black	420 (12.94)	95 (12.09)		44 (12.46)	49 (13.88)	
Other^#^	238 (7.33)	51 (6.49)		25 (7.08)	31 (8.78)	
Residence type, n (%)			0.261			0.314
Urban	2896 (89.19)	690 (87.79)		314 (88.95)	322 (91.22)	
Rural	351 (10.81)	96 (12.21)		39 (11.05)	31 (8.78)	
Year of diagnosis (%)			<0.001			0.123
2004-2015	1997 (61.50)	660 (83.97)		286 (81.02)	269 (76.20)	
2016-2017	405 (12.47)	61 (7.76)		40 (11.33)	41 (11.61)	
2018-2023	845 (26.02)	65 (8.27)		27 (7.65)	43 (12.18)	
Tumor location (%)			<0.001			0.778
Stomach	351 (10.81)	27 (3.44)		27 (7.65)	27 (7.65)	
Small Intestine	187 (5.76)	277 (35.24)		54 (15.30)	47 (13.31)	
Colon and Rectum	718 (22.11)	348 (44.27)		133 (37.68)	145 (41.08)	
Pancreas	1991 (61.32)	134 (17.05)		139 (39.38)	134 (37.96)	
Tumor size (%)			<0.001			0.795
≤50 mm	1118 (34.43)	438 (55.73)		154 (43.63)	151 (42.78)	
>50 mm	922 (28.40)	274 (34.86)		132 (37.39)	140 (39.66)	
Unknown	1207 (37.17)	74 (9.41)		67 (18.98)	62 (17.56)	
T stage (%)			<0.001			0.781
T0/T1	306 (9.42)	42 (5.34)		35 (9.92)	30 (8.50)	
T2	586 (18.05)	87 (11.07)		65 (18.41)	67 (18.98)	
T3	617 (19.00)	357 (45.42)		132 (37.39)	129 (36.54)	
T4	387 (11.92)	260 (33.08)		77 (21.81)	89 (25.21)	
Tx	1351 (41.61)	40 (5.09)		44 (12.46)	38 (10.76)	
N stage (%)			<0.001			0.563
N0	1305 (40.19)	154 (19.59)		102 (28.90)	115 (32.58)	
N1/N2/N3	833 (25.65)	597 (75.95)		216 (61.19)	206 (58.36)	
Nx	1109 (34.15)	35 (4.45)		35 (9.92)	32 (9.07)	
Radiation (%)			0.003			0.896
No	2928 (90.18)	736 (93.64)		321 (90.93)	320 (90.65)	
Yes	319 (9.82)	50 (6.36)		32 (9.07)	33 (9.35)	
Chemotherapy (%)			<0.001			0.292
No	1452 (44.72)	495 (62.98)		186 (52.69)	172 (48.73)	
Yes	1795 (55.28)	291 (37.02)		167 (47.31)	181 (51.27)	
Survival months (median[IQR])	7.00 [22.00]	24.00 [82.75]	<0.001	8.00 [21.00]	23.00 [65.00]	<0.001
Vital status (%)			<0.001			<0.001
Alive	346 (10.66)	154 (19.59)		35 (9.92)	69 (19.55)	
Dead	2901 (89.34)	632 (80.41)		318 (90.08)	284 (80.45)	
Cause-specific death status (%)			<0.001			<0.001
Alive	548 (16.88)	231 (29.39)		58 (16.43)	97 (27.48)	
Dead	2699 (83.12)	555 (70.61)		295 (83.57)	256 (72.52)	

All values are presented as median (IQR), or counts (proportion).

PSM, propensity score matching. Other^#^, American Indian/AK Native, Asian/Pacific Islander.

After 1:1 PSM, 353 patients were retained in each group. Among the 706 participants, with a median age of 61.00 years (range 21–89 years), 602 cases (85.27%) died, and 551 cases (78.05%) died from GEP-NEC. The median follow-up time was 13 months (range 0–223 months). This cohort consists of 376 males (53.26%) and 330 females (46.74%) (**Table 1**). No statistically significant differences were observed between the two groups with respect to matching covariates (all P>0.05). Except for the diagnosis year with an SMD slightly higher than 0.1, the SMDs of all other covariates were below 0.1, indicating good covariate balance after matching (**Table 1**; **Supplementary Figures S1, S2**).

### 3.2 Kaplan-Meier survival analysis

**Figure 2:**
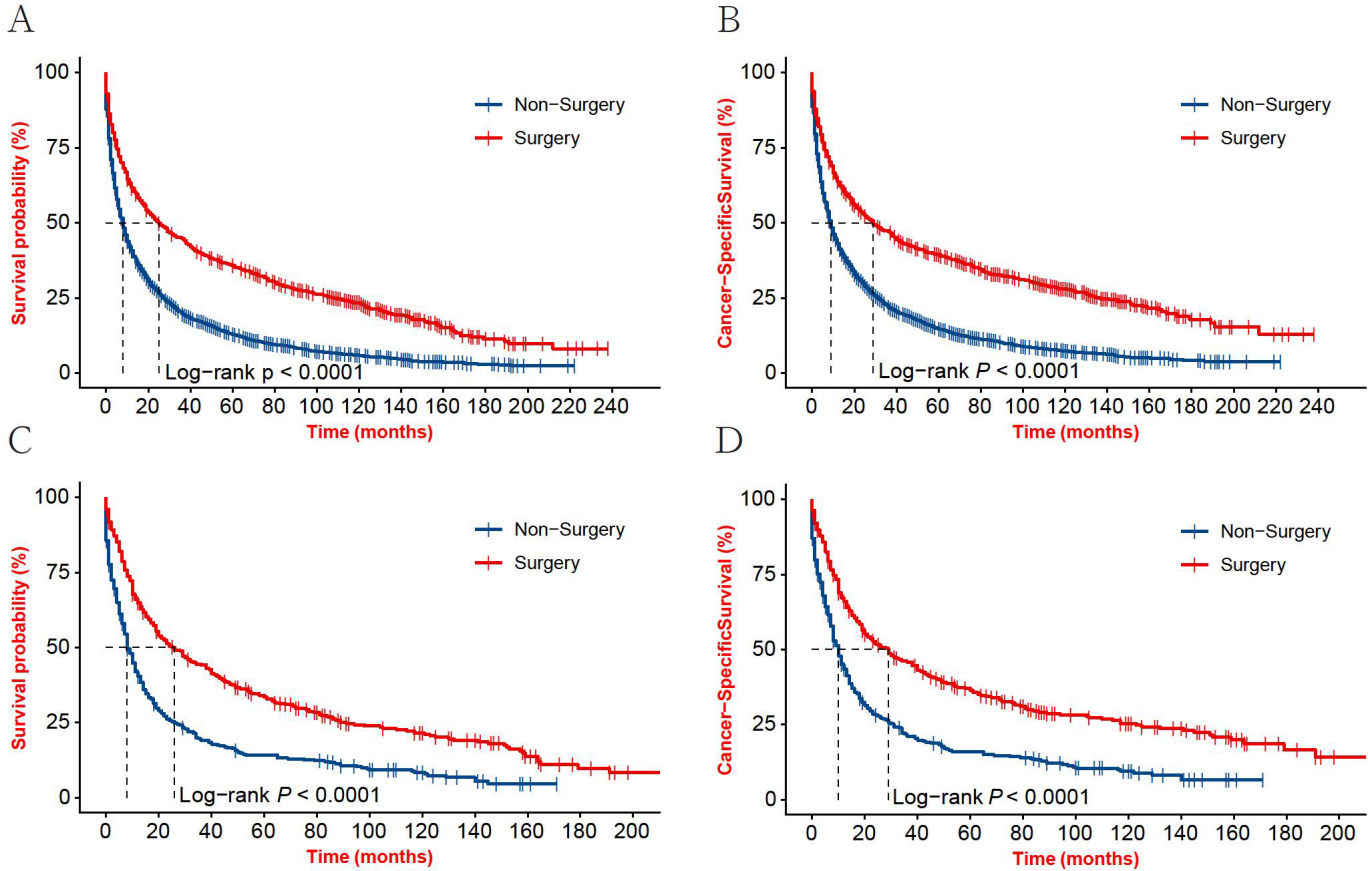
Kaplan–Meier curves for OS and CSS in GEP-NEC patients. **(A)** OS before PSM; **(B)** CSS before PSM; **(C)** OS after PSM; **(D)** CSS after PSM.

Kaplan-Meier analysis showed that the surgery group had significantly longer OS and CSS than the non-surgery group, both before and after PSM. Median OS was longer in the surgery group before PSM (25.00 vs. 8.00 months, log-rank P < 0.001) and after PSM (26.00 vs. 8.00 months, log-rank P < 0.001). Similarly, median CSS was longer in the surgery group before PSM (29.00 vs. 10.00 months, log-rank P <0.001) and after PSM (29.00 vs. 9.00 months, log-rank P <0.001; **Figures 2A–D**).

**Figure 3:**
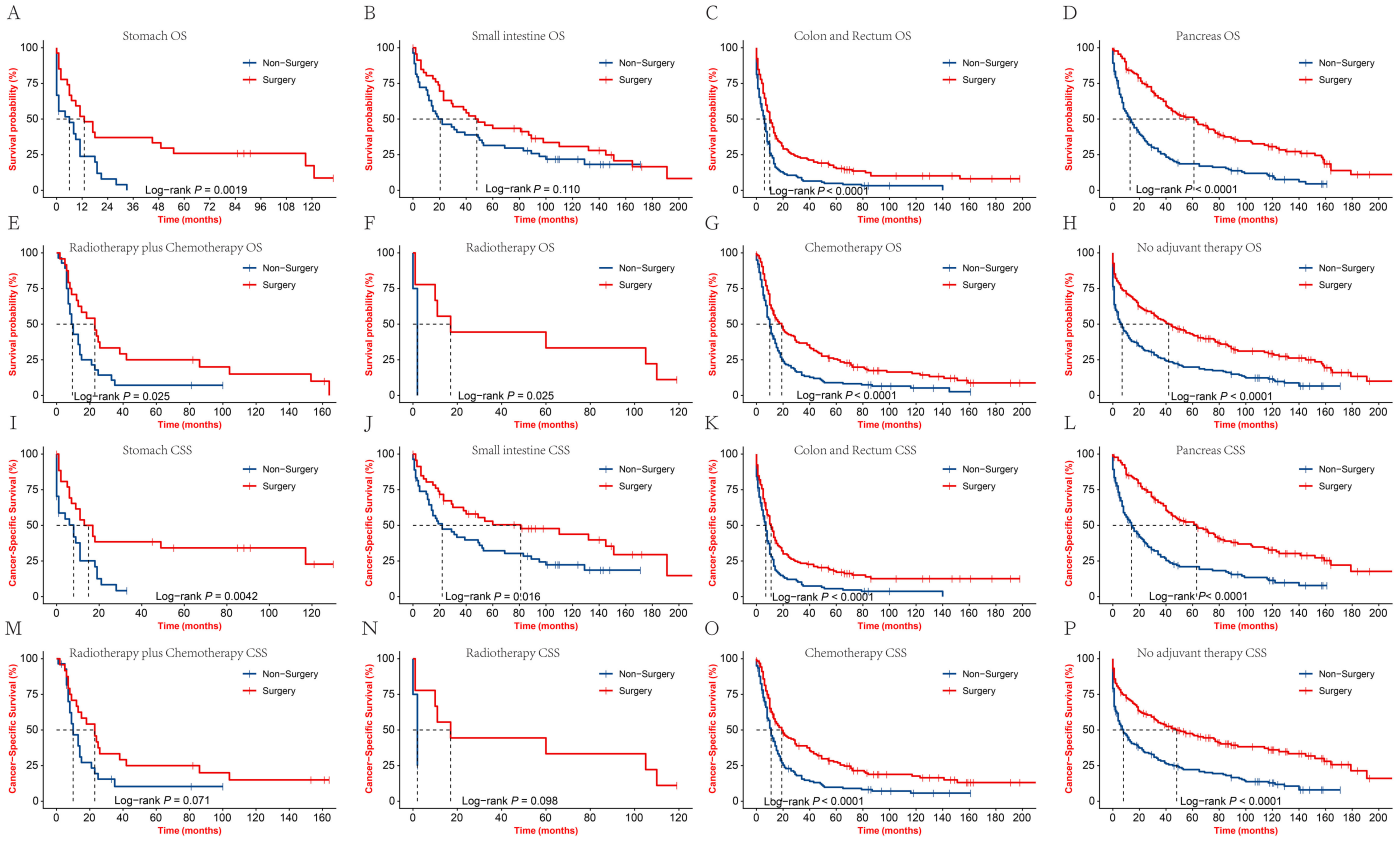
Stratified Kaplan-Meier survival curves comparing surgery versus non-surgery in the PSM-matched cohort. Kaplan-Meier curves for OS **(A–H)** and CSS **(I–P)** were stratified by primary tumor site and treatment modality. **(A–D)** OS curves stratified by primary tumor site: **(A)** gastric NEC, **(B)** small intestinal NEC, **(C)** colorectal NEC, and **(D)** pancreatic NEC. **(E–H)** OS curves stratified by treatment modality: **(E)** chemoradiotherapy, **(F)** radiotherapy alone, **(G)** chemotherapy alone, and **(H)** no radiotherapy or chemotherapy. **(I–L)** CSS curves stratified by primary tumor site: **(I)** gastric NEC, **(J)** small intestinal NEC, **(K)** colorectal NEC, and **(L)** pancreatic NEC. **(M–P)** CSS curves stratified by treatment modality: **(M)** chemoradiotherapy, **(N)** radiotherapy alone, **(O)** chemotherapy alone, and **(P)** no radiotherapy or chemotherapy. OS, overall survival; CSS, cancer-specific survival; NEC, neuroendocrine carcinoma; PSM, propensity score matching. GEP-NEC, gastroenteropancreatic neuroendocrine carcinoma; OS, overall survival; CSS, cancer-specific survival; PSM, propensity score matching.

Stratified Kaplan-Meier analyses were further conducted in the PSM-matched cohort. When stratified by primary tumor site, surgery was associated with significantly longer OS and CSS in patients with gastric, small intestinal, colorectal, and pancreatic NEC (all log-rank P < 0.05; **Figures 3A–D, I–L**). When stratified by treatment modality, surgery was associated with significantly longer OS and CSS among patients who received chemotherapy alone and those who received neither radiotherapy nor chemotherapy (all log-rank P <0.05). Among patients who received chemoradiotherapy or radiotherapy alone, the surgery group showed numerically longer OS and CSS, but the differences were not statistically significant (all log-rank P >0.05; **Figures 3E–H, M–P**).

### 3.3 Multivariable cox regression analysis of OS and CSS

Multivariable Cox regression analyses with stepwise covariate adjustment were performed to assess the association between surgery and survival outcomes in patients with GEP-NEC (**Table 2**). In Model 4, the fully adjusted model, surgery was independently associated with better OS and CSS compared with non-surgery (OS: HR = 0.47, 95% CI: 0.40-0.56, P < 0.001; CSS: HR = 0.46, 95% CI: 0.39-0.54, P < 0.001).

**Table 2:** Multivariable cox regression of surgery on OS and CSS in GEP-NEC patients.

Characteristic	OS		CSS	
	HR (95% CI)	P value	HR (95% CI)	P value
Non-surgery	Reference		Reference	
Surgery				
Model 1	0.54 (0.46 ~ 0.64)	<0.001	0.54 (0.46 ~ 0.64)	<0.001
Model 2	0.53 (0.45 ~ 0.63)	<0.001	0.53 (0.45 ~ 0.63)	<0.001
Model 3	0.52 (0.44 ~ 0.62)	<0.001	0.52 (0.44 ~ 0.61)	<0.001
Model 4	0.47 (0.40 ~ 0.56)	<0.001	0.46 (0.39 ~ 0.54)	<0.001

OS, overall survival; CSS, cancer-specific survival; GEP-NEC, gastroenteropancreatic neuroendocrine carcinoma; HR, hazard ratio; CI, confidence interval.

Model 1: The Crude adjusted model (Unadjusted).

Model 2: Adjusted for gender, age, and race.

Model 3: Adjusted for factors in Model 2 and Year of diagnosis, Residence type.

Model 4: Adjusted for factors in Model 3 and tumor location, Tumor size, T stage, N stage, Radiation, Chemotherapy.

### 3.4 Sensitivity analysis and E-value analysis

In the primary PSM-matched cohort, surgery was associated with better OS and CSS (OS: HR = 0.47, 95% CI: 0.40-0.56, P <0.001; CSS: HR = 0.46, 95% CI: 0.39-0.54, P <0.001; **Table 3**). The sensitivity analyses yielded consistent results. Surgery remained associated with longer OS and CSS in the pre-PSM cohort, the cohort excluded after PSM, the complete-case cohort, the multiple-imputation cohort, the resection-restricted post-PSM cohort, and the PSM cohort generated using a relaxed caliper of 0.2. The HRs for OS ranged from 0.41 to 0.77, and the HRs for CSS ranged from 0.41 to 0.75, with all associations remaining statistically significant (all P <0.05; **Table 3**). These findings indicate that the association between surgery and better survival outcomes was stable across different analytical strategies.

**Table 3:** Sensitivity analysis of surgery on OS and CSS in GEP-NEC patients.

Analysis strategy	OS		CSS	
	HR (95% CI)	P value	HR (95% CI)	P value
Main analysis (PSM-matched cohort) (N = 706)	0.47 (0.40 ~ 0.56)	<0.001	0.46 (0.39 ~ 0.54)	<0.001
Sensitivity analysis				
PSM-before cohort (N = 4033)	0.56 (0.50 ~ 0.62)	<0.001	0.54 (0.48 ~ 0.61)	<0.001
PSM-Excluded Cohort (N = 3327)	0.77 (0.64 ~ 0.92)	0.004	0.75 (0.62 ~ 0.91)	0.004
Complete-case analysis excluding participants with missing/unknown covariates (N = 515)	0.41 (0.33 ~ 0.50)	<0.001	0.41 (0.33 ~ 0.50)	<0.001
Multiple imputation cohort (N = 706)	0.45 (0.38 ~ 0.53)	<0.001	0.46 (0.39~ 0.55)	<0.001
Relaxed caliper PSM-matched cohort, caliper = 0.2 (N = 736)	0.47 (0.40 ~ 0.55)	<0.001	0.46 (0.39~ 0.55)	<0.001
resection-restricted post-PSM cohort^#^	0.46 (0.39 ~ 0.54)	<0.001	0.45 (0.38 ~ 0.54)	<0.001
E-value for point estimate	3.677		3.771	
E-value for CI limit closest to null	2.970		3.108	

HR, hazard ratio; CI, confidence interval; PSM, propensity score matching; GEP-NEC, gastroenteropancreatic neuroendocrine carcinoma. ^#^In the resection-restricted post-PSM cohort, the surgery group included only patients who underwent primary-site tumor resection; patients coded as tumor destruction/ablation or surgery, NOS were excluded from the surgery group.

E-value analysis was further performed to assess the potential influence of unmeasured confounding. The E-values for the associations of surgery with OS and CSS were 3.677 and 3.771, respectively. The corresponding E-values for the 95% CI limits closest to the null were 2.970 and 3.108. These results suggest that an unmeasured confounder would need to be associated with both receipt of surgery and mortality by a risk ratio of approximately 3-fold, beyond the measured covariates, to explain the observed associations fully. Therefore, the main findings showed reasonable robustness to potential unmeasured confounding (**Table 3**).

### 3.5 Subgroup analyses

**Figure 4:**
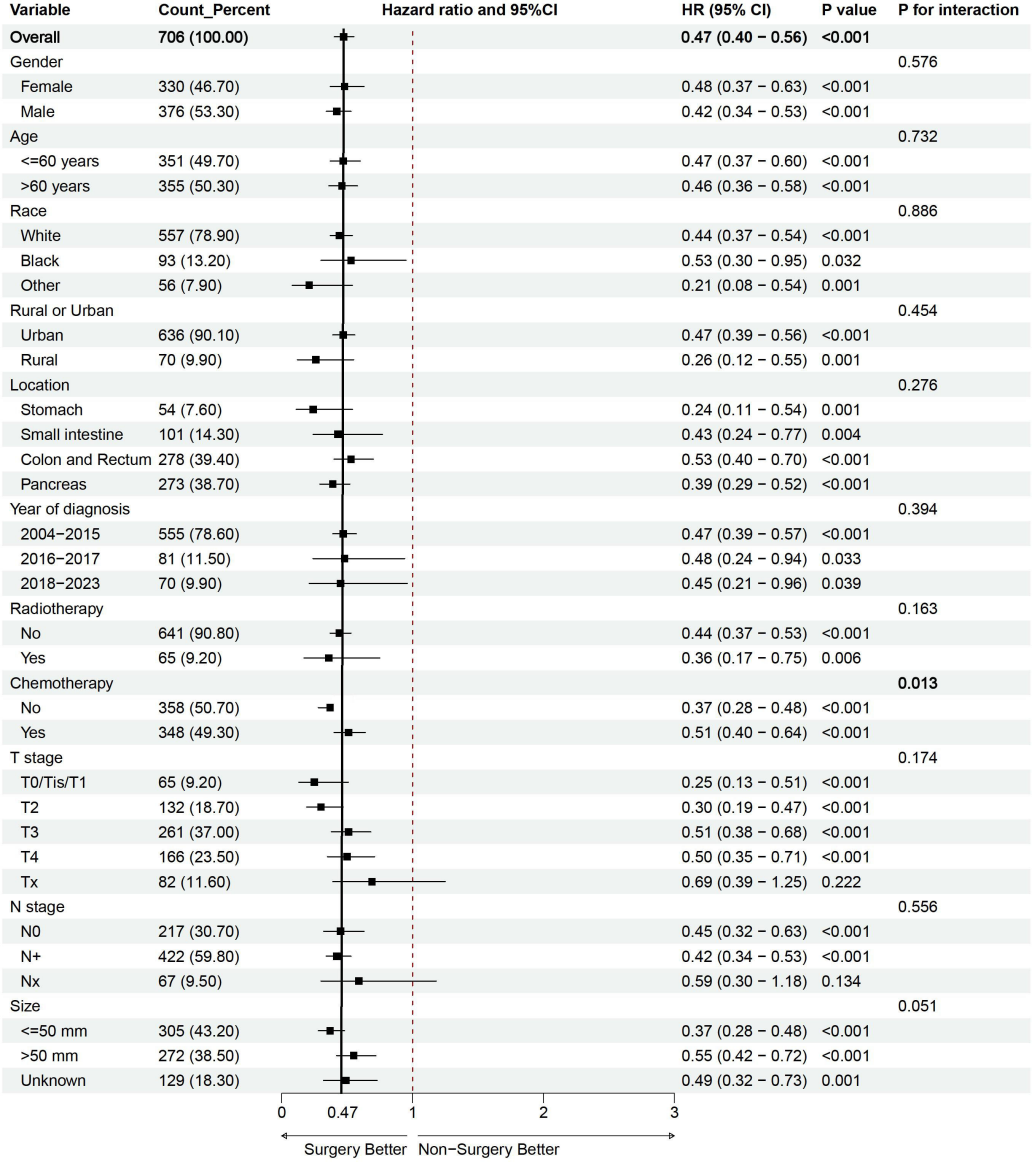
Subgroup analysis of surgery on OS in GEP-NEC patients.

Subgroup analyses were performed across all prespecified covariates. As shown in **Figure 4**; **Supplementary Figure 3**, most subgroup estimates favored surgery, although the magnitude of the association varied across subgroups. In each subgroup, surgery was associated with lower risks of all-cause mortality and cancer-specific mortality in patients with distant metastatic GEP-NEC (all P <0.05). Interaction analyses showed a significant interaction between chemotherapy status and surgery for both OS and CSS (OS: P for interaction = 0.013; CSS: P for interaction = 0.008), with stronger surgery-survival associations among patients who did not receive chemotherapy. In addition, a significant interaction was observed between tumor size and surgery in the CSS analysis (P for interaction = 0.041), with a stronger association among patients with tumors <50 mm. Subgroup analyses by diagnosis period showed similar associations between primary-site surgery and longer OS and CSS (**Supplementary Table 4**). The associations were significant in 2004–2010 and 2011–2019. In 2020–2023, the HRs remained below 1, although the 95% CIs crossed 1. No significant interactions were observed for OS (P for interaction = 0.732) or CSS (P for interaction = 0.630).

## 4 Discussion

This population-based SEER study evaluated the association between primary-site surgery and survival outcomes in patients with distant metastatic GEP-NEC diagnosed between 2004 and 2023. In the propensity score-matched cohort, patients who underwent surgery had a longer median OS than those who did not (26.0 vs. 8.0 months). A similar advantage was observed for CSS (29.0 vs. 9.0 months). In the fully adjusted Cox model, surgery was associated with better OS (HR = 0.47, 95% CI: 0.40-0.56) and CSS (HR = 0.46, 95% CI: 0.39-0.54). These associations remained consistent across multiple sensitivity analyses, and the E-value analysis suggested that strong unmeasured confounding would be required to explain the observed findings fully. Overall, these results support considering primary-site surgery as a potential component of comprehensive treatment in carefully selected patients with distant metastatic GEP-NEC.

As the understanding of NENs has evolved, it has become increasingly clear that NEC differs substantially from NET, including grade 3 NET (NET G3), in survival outcomes. Many existing studies have either combined these populations or focused primarily on NETs. Some studies of primary tumor surgery have suggested a potential survival benefit in patients with metastatic GEP-NET or GEP-NEC. However, these findings are limited by population heterogeneity and reliance on relatively outdated datasets. In a cohort of 14, 510 patients with GEP-NET, Tierney et al. reported that primary tumor resection was associated with prolonged survival across all GEP-NET subtypes (7). Wu et al. broadened the scope of exposure from tumor resection to any surgical intervention and found that surgery was associated with better OS among patients with GEP-NETs (14). In addition, a single-center retrospective study by Kivrak Salim et al. included 53 patients with G1-G2 GEP-NETs with unresectable distant metastases. Their results showed that primary tumor resection was associated with a survival advantage in advanced GEP-NETs with unresectable metastases (8). These GEP-NET-related studies provide biological and clinical rationale for the hypothesis that primary-site surgery may prolong survival.

However, as the understanding of NENs has evolved, NEC has increasingly been recognized as distinct from well-differentiated NETs, including NET G3, in biological behavior, proliferative activity, prognosis, and sensitivity to systemic therapy (15–19). Extrapolation of evidence from NET populations to NEC populations should therefore be approached with caution. Studies focusing specifically on NEC are more informative for guiding treatment decisions in this patient population. Kerekes et al. reported that, compared with no surgery, surgery involving either the primary lesion or metastatic lesions was associated with a 40% lower risk of death, whereas surgery involving both the primary and metastatic lesions was associated with a 59% lower risk of death in patients with GEP-NEC (10). Another study further narrowed the population to GI-NEC with unresectable liver metastases. It showed that primary resection significantly prolonged median OS (25.7 vs. 9.3 months) and reduced the risk of death by 57% (11). Similarly, Jiang et al. (20) reported that primary-site surgery was associated with improved CSS in patients with high-grade nonfunctional pancreatic neuroendocrine carcinoma (31 vs. 5 months). Li et al. (21) and Feng et al. (22) also observed that primary tumor resection was associated with improved OS or CSS in patients with stage IV gastric NEC and metastatic pancreatic NEC, respectively.

These retrospective studies provide evidence from different perspectives that surgery may be associated with better survival outcomes in patients with metastatic GEP-NET or GEP-NEC. In rare tumors, for which large multicenter randomized controlled trials are difficult to conduct, such real-world evidence is clinically valuable. However, changes in treatment concepts, surgical techniques, and multimodal treatment strategies have created a need for updated population-level evidence based on cohorts that better reflect contemporary clinical practice. Compared with previous studies, the present study used more recent SEER data covering patients diagnosed between 2004 and 2023. It included patients with distant metastatic GEP-NEC from multiple primary sites, including the stomach, small intestine, colorectum, and pancreas, and evaluated both OS and CSS. Appendiceal neuroendocrine carcinoma has a relatively unique biological behavior, so this study does not include the appendix. The findings showed that primary-site surgery was associated with longer OS and CSS in patients with distant metastatic GEP-NEC. This suggests that, even in the presence of distant metastasis, surgery limited to the primary lesion may still be associated with lower risks of all-cause and cancer-specific mortality.

Regarding potential mechanisms, the survival benefit associated with primary-site surgery may be multifactorial. First, surgery may reduce the overall tumor burden (23) and attenuate tumor-related inflammatory, pro-angiogenic, and immunosuppressive signaling (24–26), thereby potentially delaying systemic disease progression. Second, primary-site surgery may reduce complications such as obstruction, bleeding, perforation, and nutrition-related adverse events, which may help improve functional and nutritional status and lower the risk of complication-related death (27). Third, because patients with metastatic GEP-NEC are often treated with multimodal strategies, resection or local control of the primary lesion may create opportunities for subsequent chemotherapy, radiotherapy, or other combined treatments. Therefore, the survival benefit associated with primary-site surgery may not derive solely from palliative control of the local tumor. It may also be related to improved tolerance of systemic therapy and reduced persistent tumor-driven effects. However, these mechanisms cannot be directly validated using the SEER database. These explanations should therefore be considered plausible hypotheses rather than proven causal pathways.

The subgroup findings are clinically relevant but should be interpreted with caution. Surgery-related OS and CSS benefits were more pronounced among patients who did not receive chemotherapy. This finding is partly consistent with the study by Schmitz et al., which showed that postoperative chemotherapy after radical resection did not improve survival in patients with GEP-NEC (28). However, this should not be interpreted as evidence against chemotherapy, as systemic therapy remains an important component of treatment for advanced or unresectable NEC. A more plausible explanation is treatment selection and disease heterogeneity in real-world practice. Patients who received chemotherapy may have had more extensive metastases, higher tumor burden, or poorer performance status, and their prognosis may have been driven mainly by systemic disease progression. In this context, the relative survival benefit associated with primary-site surgery may be attenuated. In contrast, patients who did not receive chemotherapy but underwent surgery may have represented a more selected population with lower tumor burden, better local disease control, slower disease progression, or better functional status. In addition, the association between surgery and improved CSS was more pronounced in patients with tumors <50 mm. This may be related to lower primary tumor burden, less local invasion, and a greater likelihood of complete resection or effective local tumor control. However, these interpretations may be affected by selection bias and unmeasured confounding between subgroups. Because SEER does not provide sufficient information on tumor burden, performance status, resectability, or treatment sequence, these subgroup findings should be considered hypothesis-generating and require validation in future prospective cohorts.

### 4.1 Limitation

This study has several limitations. First, the study covered a long time span, during which the TNM staging system, the pathological classification of NENs, and the diagnostic distinction between NEC and NET G3 were updated. As a result, staging assessment and pathological classification may have differed across calendar periods. Although the diagnosis year was included in the multivariable models, and the period-stratified analyses yielded directionally consistent HR estimates without significant interactions, residual pathological misclassification cannot be excluded. Accordingly, our findings are most applicable to metastatic GEP-NEC identified using SEER coding and may not be fully generalizable to pathologically reviewed, poorly differentiated NEC defined according to the current WHO criteria. Second, treatment for metastatic GEP-NEC is typically multimodal. Surgery, chemotherapy, radiotherapy, and other local or systemic treatments may be combined in complex sequences. Although chemotherapy and radiotherapy were included in the models, the SEER database does not provide detailed information on chemotherapy regimens, radiotherapy dose, treatment duration, treatment response, treatment sequence, targeted therapy, immunotherapy, or other treatment modalities. Therefore, residual confounding due to differences in comprehensive treatment could not be fully controlled for. Third, similar to previous studies based on the National Cancer Database (NCDB) or single-center cohorts, this study was retrospective and observational. Although PSM, multivariable Cox regression, sensitivity analyses, and E-value analysis were used to reduce confounding, selection bias, and unmeasured confounding could not be completely avoided. Patients selected for surgery may have had better performance status, lower tumor burden, fewer comorbidities, greater resectability, or more favorable tumor biology. Therefore, the observed survival advantage cannot be attributed entirely to surgery itself. Finally, SEER lacks key clinical and pathological information, including performance status, comorbidities, exact Ki-67 values, the number and volume of metastatic lesions, liver tumor burden, local symptoms, and nutritional status. These factors may influence both surgical selection and patient prognosis. Prospective multicenter studies with detailed data on tumor burden, pathological indicators, treatment sequence, and surgical quality are therefore needed to validate these findings further.

## 5 Conclusion

Primary-site surgery was associated with longer OS and CSS in patients with distant metastatic GEP-NEC, and this association remained consistent across multiple analytical approaches. The surgery–survival association appeared stronger among patients without chemotherapy and, for CSS, those with tumors <50 mm. These findings support consideration of primary-site surgery as part of a multimodal treatment strategy for carefully selected patients with metastatic GEP-NEC. Prospective studies incorporating detailed data on tumor burden, treatment sequence, and surgical quality are warranted further to clarify the role of surgery in this disease.

## Data Availability

The datasets presented in this study can be found in online repositories. The names of the repository/repositories and accession number(s) can be found below: https://seer.cancer.gov/. The datasets used and/or analyzed during the current study are available from the SEER database or from the corresponding author upon reasonable request.
