# Supplementary material for: Association of primary-site surgery with survival in patients with metastatic gastroenteropancreatic neuroendocrine carcinoma: a population-based SEER study (2004–2023)
Source: Front Oncol. 2026 Jul 22;16:1910869. doi: 10.3389/fonc.2026.1910869 (PMC13437322; doi:10.3389/fonc.2026.1910869)
Supplement: Supplementary file 1 [file DataSheet1.docx]

**Supplementary**

**Table S1.** STROBE Checklist

|  | **Item No** | **Recommendation** | **Page No** |
| --- | --- | --- | --- |
| **Title and abstract** | 1 | (*a*) Indicate the study’s design with a commonly used term in the title or the abstract | 1 |
|  |  | (*b*) Provide in the abstract an informative and balanced summary of what was done and what was found | 1-2 |
| **Introduction** | | | |
| Background/rationale | 2 | Explain the scientific background and rationale for the investigation being reported | 3-4 |
| Objectives | 3 | State specific objectives, including any prespecified hypotheses | 4 |
| **Methods** | | | |
| Study design | 4 | Present key elements of study design early in the paper | 4-5 |
| Setting | 5 | Describe the setting, locations, and relevant dates, including periods of recruitment, exposure, follow-up, and data collection | 4-5 |
| Participants | 6 | (*a*) Give the eligibility criteria, and the sources and methods of selection of participants. Describe methods of follow-up | 4-5 |
|  |  | (*b*) For matched studies, give matching criteria and number of exposed and unexposed | 8 |
| Variables | 7 | Clearly define all outcomes, exposures, predictors, potential confounders, and effect modifiers. Give diagnostic criteria, if applicable | 5-6 |
| Data sources/ measurement | 8 | For each variable of interest, give sources of data and details of methods of assessment (measurement). Describe comparability of assessment methods if there is more than one group | 5-6 |
| Bias | 9 | Describe any efforts to address potential sources of bias | 7-8 |
| Study size | 10 | Explain how the study size was arrived at | 7 |
| Quantitative variables | 11 | Explain how quantitative variables were handled in the analyses. If applicable, describe which groupings were chosen and why | Not applicable |
| Statistical methods | 12 | (*a*) Describe all statistical methods, including those used to control for confounding | 8-9 |
|  |  | (*b*) Describe any methods used to examine subgroups and interactions | 8 |
|  |  | (*c*) Explain how missing data were addressed | 7 |
|  |  | (*d*) If applicable, explain how loss to follow-up was addressed | 4 |
|  |  | (*e*) Describe any sensitivity analyses | 8-9 |
| **Results** | | |  |
| Participants | 13 | (a) Report numbers of individuals at each stage of study—eg numbers potentially eligible, examined for eligibility, confirmed eligible, included in the study, completing follow-up, and analysed | 4, 9 |
|  |  | (b) Give reasons for non-participation at each stage | 4-5 |
|  |  | (c) Consider use of a flow diagram | 26 |
| Descriptive data | 14 | (a) Give characteristics of study participants (eg demographic, clinical, social) and information on exposures and potential confounders | 10 |
|  |  | (b) Indicate number of participants with missing data for each variable of interest | Not applicable |
|  |  | (c) Summarise follow-up time (eg, average and total amount) | Not applicable |
| Outcome data | 15 | Report numbers of outcome events or summary measures over time | 9 |
| Main results | 16 | (*a*) Give unadjusted estimates and, if applicable, confounder-adjusted estimates and their precision (eg, 95% confidence interval). Make clear which confounders were adjusted for and why they were included | 10 |
|  |  | (*b*) Report category boundaries when continuous variables were categorized | 6 |
|  |  | (*c*) If relevant, consider translating estimates of relative risk into absolute risk for a meaningful time period | Not applicable |
| Other analyses | 17 | Report other analyses done—eg analyses of subgroups and interactions, and sensitivity analyses | 10-11 |
| **Discussion** | | | |
| Key results | 18 | Summarise key results with reference to study objectives | 12 |
| Limitations | 19 | Discuss limitations of the study, taking into account sources of potential bias or imprecision. Discuss both direction and magnitude of any potential bias | 15-16 |
| Interpretation | 20 | Give a cautious overall interpretation of results considering objectives, limitations, multiplicity of analyses, results from similar studies, and other relevant evidence | 11-16 |
| Generalisability | 21 | Discuss the generalisability (external validity) of the study results | Not applicable |
| **Other information** | | | |
| Funding | 22 | Give the source of funding and the role of the funders for the present study and, if applicable, for the original study on which the present article is based. | Not applicable |

**Note:** An Explanation and Elaboration article discusses each checklist item and gives methodological background and published examples of transparent reporting. The STROBE checklist is best used in conjunction with this article (freely available on the websites of PLoS Medicine at http://www.plosmedicine.org/, Annals of Internal Medicine at http://www.annals.org/, and Epidemiology at http://www.epidem.com/). Information on the STROBE Initiative is available at [http://www.strobe-statement.org.](http://www.strobe-statement.org)

**Table S2.** SEER case-definition codes used to identify GEP-NEC

| Variable | Included categories/codes |
| --- | --- |
| Site recode ICD-O-3/WHO 2008 | Stomach; Small Intestine; Colon and Rectum; Pancreas |
| Primary Site - labeled | C16.0–C16.9; C17.0–C17.9; C18.0; C18.2–C18.9; C19.9; C20.9; C25.0–C25.9 |
| ICD-O-3 Histology/Behavior | 8246/3: Neuroendocrine carcinoma, NOS |
| Excluded primary site | C18.1 Appendix |

**Table S3.** Harmonization of SEER primary-site surgery codes before and after 2023

| Surgical category | 1998–2022 codes | 2023 codes | Analytical handling | Sample size before PSM | Sample size after PSM |
| --- | --- | --- | --- | --- | --- |
| No surgery | 00 | A000 | Non-Surgery | 3247 | 353 |
| Tumor destruction/ablation | 10–19 | A100–A190 | Surgery | 0 | 0 |
| Resection | 20–80 | A200–A800 | Surgery | 767 | 339 |
| Surgery, NOS | 90 | A900 | Surgery | 19 | 14 |
| Special/unknown | 98–99 | A980–A990 | Excluded | 0 | 0 |
| Blank | Blank | Blank | Excluded | 0 | 0 |

**Note:** According to the SEER website, beginning in 2023, the original two-digit surgery field was expanded to a four-character alphanumeric field, such as A450 or B450. This change primarily reflects the evolution of surgical techniques and terminology. However, not all site-specific surgery code groups have been reviewed. For site groups that have not yet been reviewed, all surgery codes begin with the letter “A”, followed by the two-digit code used in the 1998–2022 field, with a trailing zero appended. For example, surgery code 30 in the 1998–2022 field becomes A300 in the new field. For site groups in which surgery codes have been updated, the four-character alphanumeric codes begin with the letter “B”, such as B300. These B-prefixed codes may differ from both the original numeric codes and the corresponding A-prefixed codes. We reviewed the 2023 surgery codes for GEP-NEC and found that all relevant codes belonged to site groups that had not undergone code review/update; therefore, all observed codes were A-prefixed. For analytic consistency, we converted these A-prefixed codes to their corresponding two-digit legacy numeric codes to harmonize them with the pre-2023 surgery codes.

**Table S4.** Subgroup analysis of the association of primary lesion surgery with OS and CSS based on different WHO classification-related calendar periods

|  |  | OS | |  | CSS | |
| --- | --- | --- | --- | --- | --- | --- |
| WHO classification-related calendar periods |  | HR (95% CI) | P value |  | HR (95% CI) | P value |
| 2004-2010 | Non-Surgery (n=129) | Reference |  |  | Reference |  |
|  | Surgery (n=166) | 0.47 (0.36-0.61) | <0.001 |  | 0.48 (0.37-0.63) | <0.001 |
| 2011-2019 | Non-Surgery (n=207) | Reference |  |  | Reference |  |
|  | Surgery (n=163) | 0.43 (0.34-0.55) | <0.001 |  | 0.42 (0.32-0.54) | <0.001 |
| 2020-2023 | Non-Surgery  (n=17) | Reference |  |  | Reference |  |
|  | Surgery  (n=24) | 0.38 (0.15-1.00) | 0.051 |  | 0.44 (0.16-1.20) | 0.109 |
| P for interaction |  | 0.732 | |  | 0.630 | |

**Note:** HR, hazard ratio; CI, confidence interval; OS, overall survival; CSS, cancer-specific survival; GEP-NEC, gastroenteropancreatic neuroendocrine carcinoma.

Adjusted covariates: gender, age, and race; residence type; tumor location; tumor size; T stage; N stage; radiation; chemotherapy.

Fig. S1.The distribution of propensity scores before and after PSM. Histograms show the distribution of estimated propensity scores in the surgery and non-surgery groups before and after PSM. PSM, propensity score matching.

Fig. S2 Absolute Standardized Mean Differences plot of covariate balance.

**Note:** After PSM, covariate balance was substantially improved. All covariates except year of diagnosis achieved an SMD <0.1; the SMD for year of diagnosis was slightly above the threshold (0.15). Therefore, year of diagnosis was further adjusted for in the multivariable Cox regression models, and sensitivity analyses were performed to assess the robustness of the findings.

Fig. S3 Subgroup analysis of surgery on CSS in GEP-NEC patients. Hazard ratios and 95% confidence intervals are shown for prespecified subgroups in the PSM-matched cohort. CI, confidence interval; CSS, cancer-specific survival; HR, hazard ratio; PSM, propensity score matching.
